# Diversity of Amyloid Motifs in NLR Signaling in Fungi

**DOI:** 10.3390/biom7020038

**Published:** 2017-04-13

**Authors:** Antoine Loquet, Sven J. Saupe

**Affiliations:** 1 Institute of Chemistry and Biology of Membranes and Nanoobjects, UMR 5248 CBMN-CNRS Université de Bordeaux, Allée Geoffroy Saint-Hillaire, 33600 Pessac, France; a.loquet@iecb.u-bordeaux.fr; 2 Non-Self Recognition in Fungi, Institut de Biochimie et de Génétique Cellulaire, UMR 5095 CNRS Université de Bordeaux, 1 rue Camille Saint Saëns, 33077 Bordeaux CEDEX, France

**Keywords:** amyloid, prion, programmed cell death, NLR, filamentous fungi

## Abstract

Amyloid folds not only represent the underlying cause of a large class of human diseases but also display a variety of functional roles both in prokaryote and eukaryote organisms. Among these roles is a recently-described activity in signal transduction cascades functioning in host defense and programmed cell death and involving Nod-like receptors (NLRs). In different fungal species, prion amyloid folds convey activation signals from a receptor protein to an effector domain by an amyloid templating and propagation mechanism. The discovery of these amyloid signaling motifs derives from the study of [Het-s], a fungal prion of the species *Podospora anserina*. These signaling pathways are typically composed of two basic components encoded by adjacent genes, the NLR receptor bearing an amyloid motif at the N-terminal end and a cell death execution protein with a HeLo pore-forming domain bearing a C-terminal amyloid motif. Activation of the NLR receptor allows for amyloid folding of the N-terminal amyloid motifs which then template trans-conformation of the homologous motif in the cell death execution protein. A variety of such motifs, which differ by their sequence signature, have been described in fungi. Among them, the PP-motif bears resemblance with the RHIM amyloid motif involved in the necroptosis pathway in mammals suggesting an evolutionary conservation of amyloid signaling from fungi to mammals.

## 1. NLR in Animals and Plants and NLR-Like Receptors in Fungi

**Figure 1:**
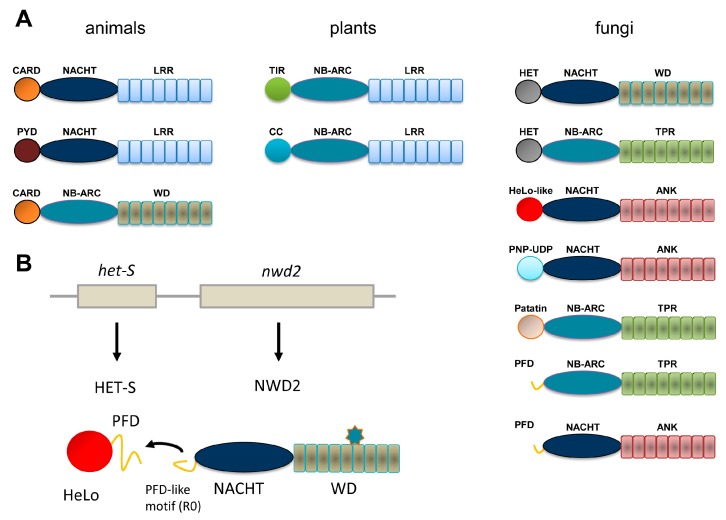
Domain organization of Nod-like receptor (NLR) and NLR-like proteins in animals, plants and fungi. (**A**) The most common domain organization for Nod-like (or NB-leucine-rich repeat (LRR)) proteins and related proteins in animals, plants and fungi are listed. The animal group includes the APAF-1 regulator of the intrinsic apoptosis pathway (bottom diagram). Only a selection of the more common domain-architectures is given. (**B**) Genome organization and domain diagram of the *nwd2/het-s* gene cluster of *Podospora anserina*. The star-shaped figure represents the cognate ligand of the NWD2 repeat domain.

Recognition and adequate response to biotic interactions are critical in immune defense and for the establishment of efficient symbioses. Immunity involves both cell surface and intracellular receptors. Plants and animals display a repertoire of intracellular receptors of the NLR (nucleotide binding domain, NBD and leucine-rich repeat, LRR) superfamily that is employed both to fight off pathogens and to regulate symbiotic interactions with micro-organisms [1,2]. Although the acronym NLR is often used as derived from the expression Nod-like receptors, originating from the animal immunity field, some authors prefer to adhere to the nomenclature given above to take into account that this type of receptors in found both in animals and in plants. NLRs display a typical tripartite domain architecture with a central NBD domain flanked N-terminally with an effector domain and C-terminally with a LRR domain. Two subclasses of NBDs are distinguished, NACHT domains are found in animals, whereas plant NLRs display NB-ARC domains. In animal NLRs, the N-terminal domains most often belong to the death-fold superfamily (including CARD and Pyrin domains) while in plants two main subtypes can be distinguished as either TIR and CC (coiled-coil) domains (Figure 1A). NLRs are activated by direct or indirect recognition of triggering cues and post-activation responses often involve induction of programmed cell death (typically in the form of a hypersensitive response in plants) but also production of pro-inflammatory cytokines in animals. The detailed mode of activation of NLRs remains poorly defined in particular in plant models. So far the APAF-1 inducer of the intrinsic apoptosis pathway generally served as a paradigm from the activation mechanism of NLRs [3] but cryoelectron tomography structures of animal NLRs start to emerge [4]. NLRs exist in an auto-inhibited form in which the LRR domain participates in the auto-inhibition, binding of a pathogen-derived ligand induces formation of an oligomerisation competent state that leads to the assembly of an active oligomer. The oligomerisation step is thought to mediate proximity-driven activation of the N-terminal effector domains. For some animal NLRs it has been shown that the pathogen-derived signal is directly recognized (for instance, NAIP5/NLRC4 recognize bacterial flagellin) [5] but more complex strategies of recognition have also been developed probably resulting from the intense arm race between host and pathogen [6]. In plants, in the guard model it is the modification of a *guardee* protein by a pathogen-effector that triggers activation. Plants also employ decoys that lure pathogen effectors and allow their detection. Such decoys can occur as independent proteins or be present as integrated decoys in the C-terminal region of NLRs. In spite, of the common features of NLRs in plants and animals, the mechanistic details in the activation mode and the post-activation responses presumably are extremely diverse consistent with the role of these receptors in immune response, a biological activity in which diversification is critical.

NLR have also been described in fungi adding an important piece to the comparative description of this type of receptors in the eukaryotic reign [7]. We chose to adhere to the NLR designation for this class of proteins in fungi in using it as a derivation of Nod-like receptors. But they are also sometimes referred to as NLR-like because in this branch LRR repeats are not found but are replaced by a variety of other superstructure forming repeats such as TPR (tetratricopeptide repeats), WD repeats or ANK (ankyrin) repeats which makes the NLR designation (understood as NB-LRR) unsuited. In contrast, to what is found in animals and plants, NBDs can be either of the NB-ARC or NACHT type (Figure 1A). There is a large diversity of effector domains, many of which still lack a functional annotation. Twelve main types have been identified, some of which correspond to domains with a predicted enzymatic activity in contrast to TIR and death-fold family domains which function as adaptor domains. NB-ARC and NACHT domains can be found in almost all combinations with the various types of N-terminal effector domains and WD, ANK or TPR domains, suggesting that the different domain architectures arise from combinatorial associations between elementary building blocks. WD, ANK and TPR repeats of fungal NLR can display high levels of internal conservation and be subject to repeat copy number polymorphism and concerted evolution between repeats. A fraction of the NLR repertoires found in fungal genomes display an original gene and domain architecture. In this class of NLRs, a typical effector domain is not found but instead is encoded by the gene immediately adjacent in the genome [8]. In place of the effector domain, a short amyloid sequence motif is found and the same motif is also found as a C-terminal extension in the effector domain encoded by the adjacent gene. Activation of the effector domain is not the direct result of the NLR oligomerization but rather is mediated by transmission of the amyloid fold from the receptor to the effector protein. This mode of signal transduction was first identified in the NWD2/HET-S system and further generalized to other NLR and effector protein pairs [8,9,10,11] as will be described herein (Figure 1B). Description of this mode of amyloid signaling stems from the study of a fungal non-self recognition phenomenon known as heterokaryon incompatibility which was later found to derive from a NLR-based signaling cascade. The description of this system offers a paradigm to frame the current understanding of other amyloid signaling modules in fungi.

## 2. The HET-s/HET-S System

**Figure 2:**
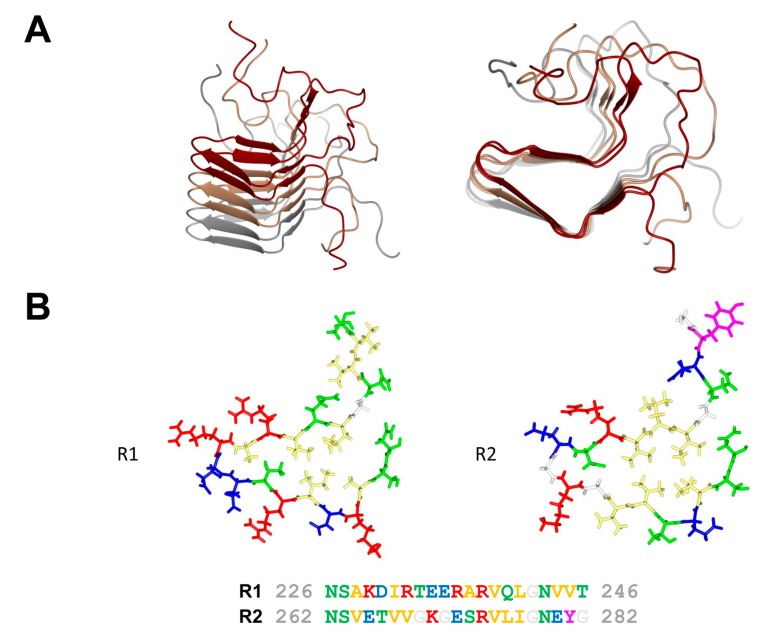
Amyloid fold of the HET-s prion forming domain. (**A**) Lateral and axial view of a trimer of HET-s (218–289) in its fibrillar amyloid conformation. Each monomer is given in a different color (PDB entry 2RMN). (**B**) Structure of the R1 and R2 repeats of HET-s (218–289) given together with the corresponding amino acid sequence. Polar residues are colored in green, hydrophobic residues in yellow, positively charged residues in red, negatively charged residues in blue, aromatic residues in magenta and glycine in white.

In fungi, when different isolates of the same species fuse, most often a cell death reaction ensues. This phenomenon known as heterokaryon incompatibility is controlled by specific loci termed heterokaryon incompatibility genes (*het* genes) [12]. In the species *Podospora anserina,* the *het-s* (small s) and *het-S* (large S) genes form a pair of incompatibility genes. Fusion between a *het-s* and a *het-S* strain leads to cell death [13]. The specific characteristic however of this incompatibility system is that the *het-s*-encoded protein is a prion protein. The *het-s* strains exist in two distinct epigenetic states termed [Het-s*] and [Het-s], in [Het-s*] strains the protein is soluble and monomeric while in [Het-s] strains in assembles into prion aggregates. Importantly incompatibility occurs when the prion form of HET-s interacts with HET-S. HET-s is a two domain protein, with a N-terminal globular domain termed HeLo and a C-terminal prion forming domain (PFD). The C-terminal prion forming domain (HET-s (218–289)) is necessary and sufficient for prion propagation and has been extensively used as a model system for studying the structure-function relation in amyloid formation and prion propagation. HET-s PFD fibrils show no evidence of structural polymorphism as they lead to very well-resolved solid state nuclear magnetic resonance (NMR) spectra. The HET-s PFD prion fibrils form a β-solenoid structure with two rungs of β-strands per monomer and comprising two 21 residue-long imperfect repeats connected by a 15 amino acid flexible loop [14] (Figure 2). Each of pseudo-repeats forms one 4.7 Å layer in the cross-β structure. Each repeat is composed of four β-strands delimiting a hydrophobic core. At the C-terminal end, a semi-hydrophobic pocket is formed by an aromatic loop that folds back into a groove delimited by the third and fourth β-strands. The structure is stabilized by two asparagine ladders and three salt bridges resulting from the stacking of the two rungs of β-strands. The core is composed of hydrophobic residues and of two hydroxyl residues forming a hydrogen bond between the two layers inside the core (T233/S273). β-arc positions between the third and the fourth β-strand in both layers are occupied by conserved glycine residues. These structural elements (N-ladders, salt-bridges, hydrophobic core, buried polar residues and β-arc glycines) contribute to various extends to the prion function, β-solenoid fold and fibril stability and fibril formation rate [15,16].

HET-S differs from HET-s by 13 residues and also comprises a HeLo domain and a C-terminal prion forming domain (however as will be developed below, HET-S in contrast to HET-s cannot form a prion) [17,18,19,20]. When HET-s in the prion form interacts with HET-S, the prion forming region is templated into the β-solenoid fold and this structural transition induces in turn a conformational change in the HeLo domain which becomes activated and acquires a pore-forming activity which relies on the formation of a N-terminal transmembrane helix [19]. In vivo, upon interaction with [Het-s], the HET-S protein relocates to the cell membrane, compromises cell integrity and triggers cell death. HET-s and HET-S functionally differ in their HeLo domain, a substitution at position 33 deprives HET-S of pore-forming activity. In a sense HET-s can be viewed as a mutant form of HET-S devoid of cell death inducing activity, it is that very property (lack of toxicity associated with the amyloid form) that allows the protein to propagate as a prion in vivo [21].

## 3. The NWD2/HET-S Paradigm

In a search for sequences homologous to the HET-s PFD, it was realized (with some surprise) that the protein encoded by the gene immediately adjacent to *het-S* in the *P. anserina* genome contains at its very N-terminus a region of homology to the elementary 21 amino acid repeat forming a single rung of the β-solenoid [8]. This protein is termed NWD2 and corresponds to a NLR with a central NACHT domain and a C-terminal WD repeat domain. It was then proposed that HET-S in fact constitutes the effector protein activated by the NWD2 NLR (Figure 1B). Because the cognate ligand of NWD2 is currently unknown chimeric variants of NWD2 responding to defined ligands were constructed. Such chimeric NWD2 variants efficiently induces [Het-s] formation in a ligand-dependent manner [10]. The N-terminal region of NWD2 is both necessary and sufficient for this prion inducing activity in vivo; this region was designated R0 by reference to the R1 and R2 repeats in the HET-s PFD. A synthetic NWD2 (3–23) peptide corresponding to the R0 region assembles into amyloids with prion infectivity and represents the smallest molecular entity with prion infectivity. Solid-state NMR analyses of NWD2 (3–23) fibrils with residue-specific labelling support that R0 adopts a HET-s-like fold. It is proposed that ligand-induced oligomerisation of NWD2 allows for spatial clustering of the R0 regions leading to cooperative nucleation of the β-solenoid fold that can then template HET-S PFD transconformation and HET-S activation. NWD2/HET-S is thus a two-component system in which the signal transduction process from NLR to effector domain involves propagation of an amyloid prion fold. Note that the incompatibility reaction between [Het-s] and HET-S occurs independently of NWD2. Incompatibility is proposed to derive by exaptation from the NWD2/HET-S system, yet it does not require NWD2 to occur [21]. Remarkably, it was reported that the N-terminal region of NWD2 and the HET-s PFD can functionally substitute for the Pyrin signaling domains in the human NLRP3 NLR and ASC, its downstream effector. NWD2/HET-S can serve as a model for the understanding of other NLR/effector pairs functioning through amyloid signaling [22].

## 4. Additional Het-s-Related Amyloid Motifs

**Figure 3:**
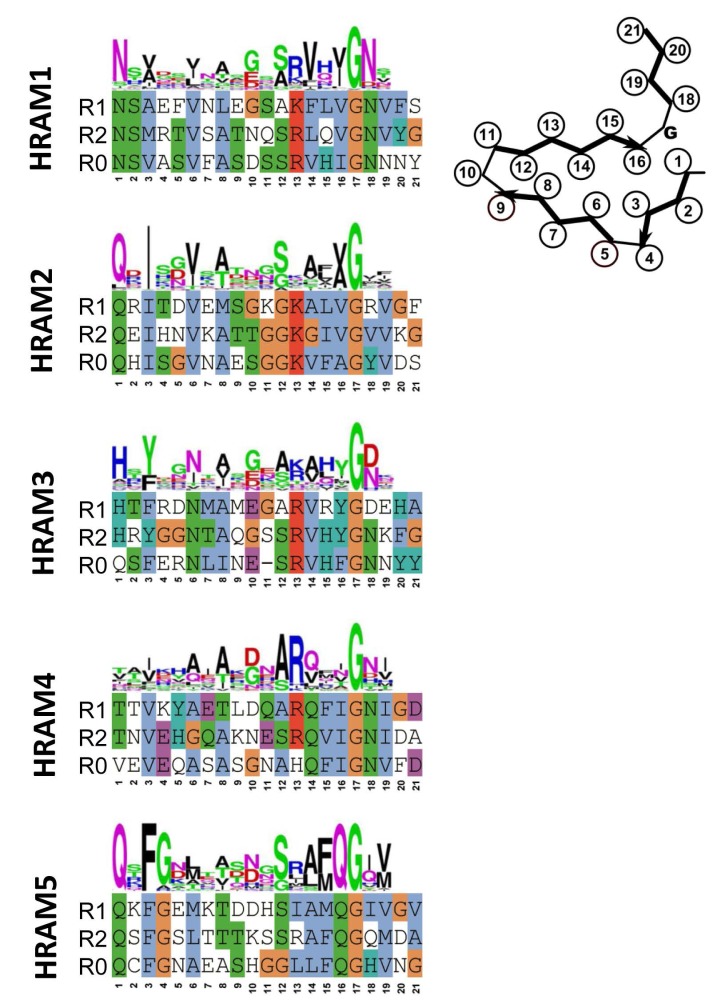
Sequences and motif signatures of the different Het-s-related-amyloid motifs (HRAM) subfamilies. For each of the five HRAM subfamilies, for one representative example, sequences of the R1, R2 and R0 sequences are given as well as the general motif signature (given as a consensus sequence logo). The proteins chosen as examples are respectively: *Fusarium graminearum* I1S1J5 and I1S1J6 for HRAM1, *Penicillium roqueforti* W6Q0M1 and W6PZN6 for HRAM2, *Cladophialophora psammophila* W9WR11 and W9XJW2 for HRAM3, *Pestalotiopsis fici* W3WR35 and W3WR59 for HRAM4 and *P. anserina* B2B1E9 and A0A090CH31 for HRAM5.

Fungal genomes were screened for motifs occurring at the C-terminus of proteins with HeLo and HeLo-like domains. In an ensemble of 579 proteins, 155 were found that harbored two repeats related in sequence to the 21 amino acid elementary HET-s-motif. In most cases, the gene adjacent to the gene encoding the HeLo (or Helo-like) domain protein did encode a NLR with at its very N-terminus a further copy of the motif. Clustering analysis of the sequences revealed the existence of 5 subfamilies that were termed HRAMs (for Het-s-related amyloid motifs) [9]. Each subfamily is defined by a specific sequence signature and in spite of considerable variation among the motif signatures, features such as presence of hydrophobic residues at positions 3, 6, 8 and 14 of the motif and presence of a G in position 17 are common to all subfamilies (Figure 3). Co-variance analyses strongly suggest that the additional HRAM subfamilies adopt a fold that is related to the *P. anserina* HET-s fold. In particular, co-variance analyses predict in-register interactions between the R1 and R2 motifs. It appears that the Het-s amyloid signaling motif has diversified into discrete subfamilies. Interestingly, in certain species as for instance *P. anserina*, HRAMs belonging to different families co-exist, opening the possibility for the occurrence of several parallel amyloid signaling pathways. Structural approaches are now required to determine to which extend the fold of these distinct HRAM families resemble the canonical HET-s fold and to which extend the different motifs do or do not cross-seed.

## 5. PP and Sigma, Two Other Types of NLR-Associated Amyloid Signaling Motifs

**Figure 4:**
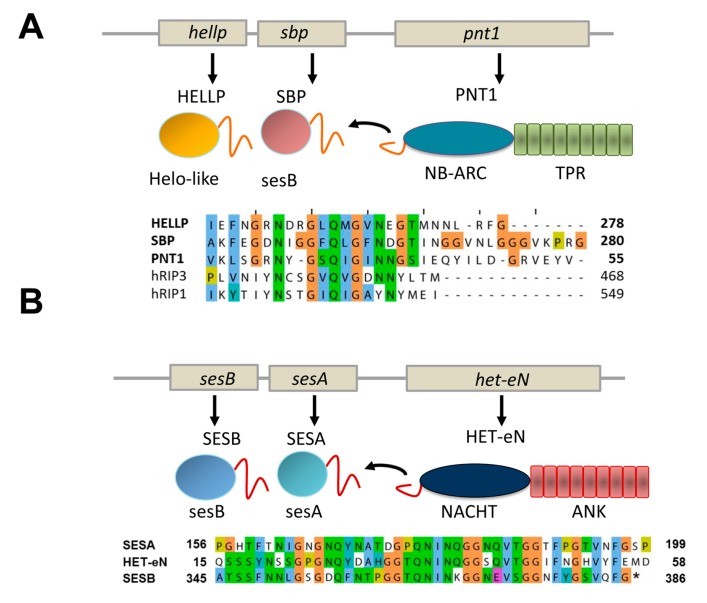
PP and sigma amyloid signaling motifs. (**A**) The gene architecture of the *hellp* locus of *Chaetomium globosum* is given as well as the domain architecture of the corresponding proteins. The sequence of the PP regions of HELLP, SBP and the PNT1 NLR is given. (**B**) The gene architecture of the *ses* locus of *Nectria haematococca* is given as well as the domain architecture of the corresponding proteins. The sequence of the sigma regions of SESA, SESB and the HET-eN NLR is given.

In addition to the HET-s motif and the related HRAM subfamilies, at least two other types of NLR-associated amyloid signaling motifs occur in fungi [8,11]. First, a motif termed PP was found to regulate activation of a HeLo-like cell death inducing protein, termed HELLP and a putative lipase termed SBP. The PP-motif is characterized by a pseudo-palindromic sequence signature centered on a conserved Q residue (Figure 4A). The motif is about 20 amino acids in length. Importantly, this motif resembles the RHIM amyloid motif (for instance by the presence of a common G×Q×G segment), found to control assembly of the RIP1 and RIP3 kinases in the necroptosis pathway in mammals [23]. The similarity between the two motifs could either represent evidence for a long term conservation of an amyloid signaling motif from fungi to mammals or alternatively result from convergent evolution of motifs unrelated from an evolutionary point of view. The PP-motif allows for activation of the HELLP cell death inducing protein which shows homology to the N-terminal 4HB domain of MLKL, the cell death execution protein in mammalian necroptosis. The RHIM motif allows for formation of the RIP1/RIP3 kinase complex which phosphorylates the C-terminal pseudo-kinase domain of MLKL [24]. Thus, while both the PP and RHIM amyloid motifs are involved in the signaling pathway leading to necroptotic or necroptosis-like cell death, they do not occur at equivalent position in the pathway in mammals and fungi. Of note is the fact that a link between fungal cell death controlling amyloids and the RHIM motif had been previously proposed [25]. Kajava et al. reported a similarity between RHIM motifs and the HET-s elementary repeats. This observation would suggest in turn that the PP and the HET-s motifs are also evolutionarily related. Future studies on the structural characterization of these motifs will probably help clarifying the evolutionary relations between these different amyloid signaling motifs.

PNT1, the NLR with PP-motif appears to control simultaneous activation of two distinct effector proteins instead of a single one as in the case of the HET-s-motif. The same situation also occurs for the sigma motif originally identified in *Nectria haematococca* [8,26]. The motif was identified in a gene cluster controlling formation of a cytoplasmic infectious element termed σ and found in three proteins, a putative NLR termed Het-eN, a predicted lipase (SESB) and a protein termed SESA (which shows a remote homology to both HeLo and Helo-like domains) (Figure 4B) [7]. The sigma (σ) motif is about 40 amino acids in length and was found to exist as different variants composed of the three-fold repetition of two submotifs [8]. 

## 6. Potential Advantages of Amyloid Signaling

**Figure 5:**
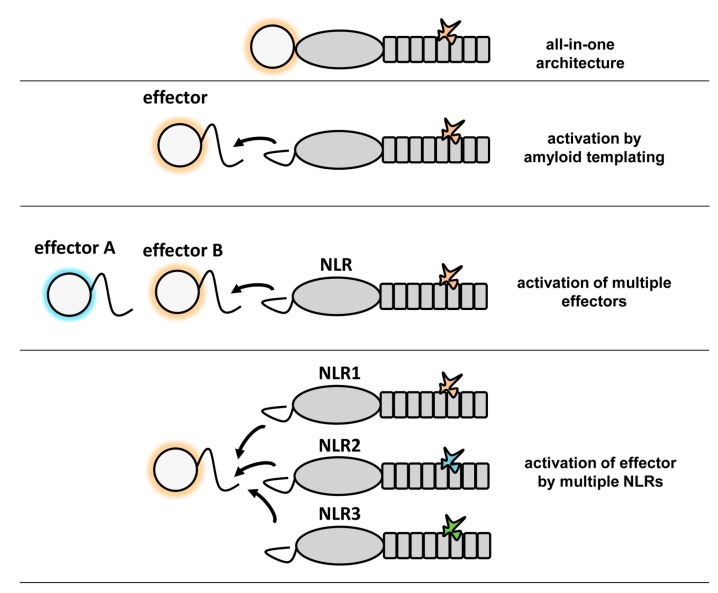
The different effector domain activation modes in fungal NLRs. The different domain architecture found in fungal NLR are given. The repeated boxes represent the superstructure-forming repeat domains (either ANK, TPR or WD), the central oval, the NBD (either NB-ARC or NACHT), the circle the effector domains (HeLo, HeLo-like, sesA, sesB, PNP-UDP phosphorylase, etc.).

The formation of higher-order signaling machines appears to be a common scheme in cellular pathways controlling cell fate and immunity [27,28]. Among such complexes are the apoptosome [3], the Myddosome [29], the MAVS CARD filaments [30], the RIP1/RIP3 necrosome [23] and the ASC-dependent inflammasomes [31]. In many cases, these signaling complexes form large filamentous assemblies. In these macromolecular complexes, different domains or motifs that have the ability to self-assemble into higher-order oligomers are employed such as the death domain fold family (DD), CARD and PYD domains, the TIR domain or an amyloid motif in the case of RHIM. Due to this capacity to self-assemble, the CARD and PYD domains were found to be able to behave as prion-forming domains in yeast [22,30]. Thus, the prion-like polymerization and propagation process in these signaling machines involves in some instances, but not in others, amyloid structures and is conserved from fungi to humans. Such higher-order assemblies whether they are based on amyloids or folded domains function by nucleated polymerization, a principle that entitles signal transduction cascades with specific properties such as signal amplification, threshold response and noise reduction [27]. The remarkable similarity between the CARD and PFD mediated signaling in NLRs has been illustrated by the fact that the HET-s prion forming motif can functionally replace the PYD domains in mammalian NLRP3 and ASC [22,28]. Self-assembly of folded globular domains or amyloid templating appear as two possible solutions for the same task of mediating an efficient transmission of a cell fate signal in an all-or-none fashion. The strictly cooperative nature of amyloid folding and the robustness of its templating and propagative properties, as well as extreme compactness make amyloid motifs well suited for such signal transduction tasks. These amyloid motifs are small and typically shorter than segments that permit formation of folded globular domains. The fact that several different motifs have been identified in fungi suggests that this mechanism shows an evolutionary success in this branch. Apparently, this mode of signal transduction has been diversified into various classes allowing for creation of versatile templating modules that can co-exist in the same cell much in the same way as diversification of the CARD domain allows several parallel signaling cascades to employ self-assembly of variants this domain. It should be noted that for each combination of effector domain and NLR-type that has been characterized to date in fungi, the same domain association can also be found in a single NLR. For instance, while in *P. anserina* the HeLo/NACHT–WD organization is found in the HET-S/NWD2 pair, in many other species display NLRs with an all-in-one HeLo–NACHT–WD architecture. The converse is so far not true as certain domain associations (such as Patatin–NB–ARC–TPR) have not been found yet in the two-gene architecture. What then could be the potential advantage of the two-gene architecture as opposed to the simpler all-in-one structure? One obvious possibility as discussed above is that the amyloid-mediated signaling allows for signal amplification. Another possible advantage, is that this mode of activation allows a single NLR to activate two distinct effector domains (as occurs for instance with the PP motif in HELLP and SBP) [11] or a single effector to be activated by different NLRs (Figure 5). 

## 7. Conclusions and Future Prospects

Fungi display large repertoires of proteins resembling plant and animal NLRs some of which have been found to be involved in non-self recognition and control of cell fate [10,11,32,33,34]. Rather than displaying LRR repeats, fungal NLR-related protein display other types of super-structure forming repeats such as ANK, TPR or WD-repeats. A fraction of these NLRs induce activation of effector domains by an amyloid templating mechanism. The *nwd2/het-S* gene pair of *P. anserina* represents the paradigm of this type of amyloid signaling mechanism but several additional amyloid signaling motifs such as the diversified HRAM family, the PP and the sigma motif have been described. These motifs appear related to the RHIM amyloid motif described in mammals and controlling the necroptosis pathway. It will now be of great interest to structurally characterize these novel motifs to see to which extent they are structurally related to the Het-s prion motif and to get a better understanding of the structural diversification of this evolutionarily successful mode of signal transduction.
